# Comparative analysis of seven types of phosphate transporters in forty species from the phylogenetic and transcriptomic perspective

**DOI:** 10.1371/journal.pone.0349574

**Published:** 2026-05-21

**Authors:** Junyi Ren, Lulu Xie, Fu Li, Siyuan Zhang, Jianchang Gao, Bin Li

**Affiliations:** 1 College of Horticulture, Shanxi Agricultural University, Taigu, Shanxi, China; 2 State Key Laboratory of Vegetable Biobreeding, Institute of Vegetables and Flowers, Chinese Academy of Agricultural Sciences, Beijing, China; National Taiwan University, TAIWAN

## Abstract

Acquisition of phosphorus (P), an essential macronutrient for plants, is a limiting factor in most soils. Phosphate transporter proteins (PTs) play an indispensable role in inorganic phosphate (Pi) transport across the membrane barriers in plants. To understand the mechanisms associated with the P use efficiency of plants, we performed a comprehensive analysis of seven types of phosphate transporters—Pi-H + co-transporters [PHT1, PHT2, PHT3, and PHT4], plastid phosphate transporters (pPTs), glycerol-3-phosphate permeases (G3Pps), and SPX domain-containing PTs—across 40 species, from bacteria to higher plants. Using the amino acid sequences of PTs obtained by BLASTp, phylogenetic and structural analysis was performed. Analysis of conserved motifs within and between types of PTs was performed using the MEME program, and a pairwise genetic distance matrix was calculated in MEGA. Moreover, transcriptomic expression patterns in five tissues (root, stem, leaf, flower, and fruit) or in P starvation were examined in eight vegetable species using public databases. In total, 564 PHT1s, 94 PHT2s, 134 PHT3s, 228 PHT4s, 116 G3Pps, 546 pPTs, and 480 SPX-domain PTs were identified. The presence or copy number of different PT types varied among taxa, indicating that PHT3s, pPTs, and SPX-domain PTs are of eukaryotic origin, whereas PHT1s, PHT2s, PHT4s, and G3Pps are of prokaryotic origin. The absence of common motifs between the various PT types suggests that they originated and diversified independently. PHT1, G3Pp, and SPX-domain PT genes showed tissue-specific and P starvation induced expression, and thus are likely candidates for genetic modification strategies to obtain P-efficient crops.

## Introduction

P is an essential macronutrient for optimal growth and development of plants. It is a fundamental element of nucleic acids and phospholipids and a key component in energy metabolism, signal transduction, and enzymatic reactions. However, acquisition of P is frequently a limiting factor in most soils. The primary forms in which P is taken up by plant roots are orthophosphates (Pi), H_2_PO^4-^, and HPO_4_^2-^, which rarely exceed 10 µM in soil solutions even in the more fertile soils [1]. The remaining quantity of phosphate ions coprecipitate with metals such as Ca, Fe, and Al, becoming unavailable for plants [2]. To improve the availability of Pi, phosphate fertilizers have been used excessively in agricultural practices in recent years. However, this activity is not environmentally friendly because of the quickly diminishing P supplies and water eutrophication. Therefore, understanding the Pi use efficiency (PUE) of plants is an urgent matter [3].

Pi transportation from soil to cells occurs in three main steps: uptake into living root cells, translocation at the whole-plant level, and homeostasis at the cellular level [4]. PTs play an indispensable role in Pi transport across the membrane barriers and against the steep concentration gradient during these processes. Since the first high-affinity phosphate transporter PHO84 in yeast was reported more than thirty years ago [5], an increasing number of its homologs as well as other proteins with phosphate transport activity, with diverse localizations in organs and subcellular compartments, have been identified [6].

Plant Pi-H + co-transporters are classified into four groups: PHT1, PHT2, PHT3, and PHT4. Proteins of the PHT1 group localize to the plasma membranes and play a role in taking up Pi from the soil and mobilizing Pi within the plant [7]. In many plants, the number of proteins in the PHT1 group is larger than that in the other groups [5]. PHO84 and the first published Arabidopsis PTs all belong to this group [6]. In Arabidopsis, AtPHT1;1 and AtPHT1;4 participate in Pi acquisition from the rhizosphere into the root, whereas AtPHT1;8 and AtPHT1;9 are likely to act sequentially in the interior of the plant during the root-to-shoot translocation [5]. Variation in Pi uptake activity among Arabidopsis accessions has also been associated with PHT1;1, supporting the central role of PHT1-mediated Pi acquisition in both controlled and natural genetic backgrounds [8]. Groups PHT2/3/4 consist of proteins that transport Pi through organelle membranes and are localized to plastid (PHT2/4), mitochondrial (PHT3), or Golgi (PHT4) membranes [9]. Plastid-localized phosphate transporters are also linked to broader plastid metabolic processes, including photosynthesis-related metabolism and carotenoid accumulation, indicating that organellar Pi transport contributes to metabolic coordination beyond Pi allocation alone [10]. Unlike PHT1/2/3, PHT4s exhibit lower specificity. They target diverse organelles, and some of them may have substrates other than Pi [11].

Vacuoles serve as a temporary reservoir for Pi ions. VPT1 (also named PHT5;1), which localizes to the tonoplast and mediates vacuolar Pi influx, has been recently functionally validated [12,13]. Its rice homolog, OsSPX-MFS3, is proposed to export Pi along the proton gradient [14]. Arabidopsis PHO1, which localizes to the Golgi and trans-Golgi network, was reported to be an exporter responsible for efflux of Pi out of pericycle cells and into the xylem vessel; thus, it transports Pi from root to shoot [15]. VPT1 and AtPHO1 belong to the SPX (SYG1/PHO81/XPR1) domain-harboring protein family. The SPX helical bundles are proposed to provide a positively charged ligand-binding surface to sense inositol polyphosphate signaling molecules, the concentrations of which change in response to Pi availability [16]. In plants, SPX-containing PTs belong to one of four subfamilies: SPX (containing only the SPX domain), SPX-EXS (ERD1/XPR1/SYG1), SPX-MFS (Major Facilitator Superfamily), and SPX-RING (Really Interesting New Gene). AtPHO1 is an SPX-EXS type protein [17].

Organellar Pi homeostasis is maintained by remobilizing Pi between plastids and the cytosol. In plastid envelope membranes, there are specific transporters that are involved in facilitating a counter-exchange of Pi and different phosphorylated carbon substrates. These pPTs are classified into the groups TPT, PPT, GPT, and XPT: TPT proteins accept triose-phosphates (TP) and 3-phosphoglycerate (3-PGA); GPT proteins accept glucose-6-phosphate (Gluc-6-P), TP, and 3-PGA; XPT proteins accept TP, xylulose-5-phosphate (Xul-5-P), ribulose-5-phosphate (Ru5-P), and erythrose-4-phosphate (Ery-4-P); and PPT proteins mediate the import of phosphoenolpyruvate (PEP) [18].

Although a readily mobile nutrient within plants, organic P derived from the breakdown of phospholipids is transported from source to sink tissues under the conditions of P starvation. The glycerol-3-phosphate permease (G3Pp) gene family participates in this process. In Arabidopsis, differentially regulated expression of all AtG3Pp genes was observed to be associated with lipid remodeling [9]. AtG3Pp1 and AtG3Pp2 were induced only in roots, whereas AtG3Pp3 and AtG3Pp4 were evident in both shoots and roots [19]. Similarly, a transporter in yeast, Git1p, mediates the uptake of extracellular glycerophosphoinositol (GroPIns) from the medium in a nutrition-dependent manner [20,21].

As one of the most basic biological activities, P uptake and translocation is precisely regulated by developmental and environmental signals. While prokaryotes facilitate their own metabolism within single cells, complex flowering plants need to ensure the allocation of nutrients to the whole body. Some PTs are constitutively expressed in different tissues, whereas others show diverse expression patterns among different tissues [22]. Moreover, the expression of many PTs may be induced in the shoot or root under low P supply, as an essential mechanism to deal with P starvation [5,6].

In the present study, we performed a comprehensive analysis to obtain an evolutionary landscape of phosphate transporters among plants, fungi, and bacteria. Moreover, transcriptomic expression patterns in five tissues or in P starvation conditions were examined in vegetable species. On the basis of this study, further analysis may be performed to study the mechanisms associated with increasing PUE.

## Materials and methods

### Identification of phosphate homologs

The genomic coding sequence (CDS) of PTs was downloaded for 40 species from Phytozome (http://www.phytozome.net/), Ensembl Plants (http://plants.ensembl.org/index.html), TAIR (http://www.arabidopsis.org/), BRAD (http://brassicadb/brad/), CuGenDB (http://icugi.org/), and NCBI (https://www.ncbi.nlm.nih.gov/genome/) databases (S1 Table).

Reference sequences covering seven types of phosphate transporters of *Arabidopsis* that have been characterized in the literature [20,23–28] (S2 Table) were used as queries to search these selected genomes using BLASTp [29]. The resulting hits were filtered by E-value (≤1e^-5^).

### Phylogenetic analysis of amino acid sequences

Amino acid sequences were aligned using MAFFT [30] and then manually curated to remove sequences with truncated conserved regions, and a total of 2162 PT sequences were retained for phylogenetic analysis (S3 Table). The best-fit amino acid substitution model was selected by MEGA [31].A maximum likelihood (ML) tree was constructed using RAxML [32] with 100 bootstrap replicates and with models WAG + G + I for PHT1s, rtREV + G for PHT2s, JTT + G for PHT3s, WAG + G for PHT4s, WAG + G for G3Pps, JTT + G + I for pPTs, and JTT + G + I for SPX-domain PTs. A neighbor-joining (NJ) tree of reference sequences was constructed in MEGA, with a p-distance model and 100 bootstrap replicates.

### Transcriptome analysis

Expression data for different tissues (root, stem, leaf, flower, and fruit) and treatments (-Pi, + Pi) were obtained from public databases (Supplemental Table 1). RNA-seq read data were first filtered using the NGS QC toolkit [33] and then mapped to reference genome sequences by Hisat2 [34]. FPKM values were calculated and normalized by the Cuffquant and Cuffnorm pipelines in Cufflinks [35].

To compare the abundance of transcripts between species, log2-transformed expression values were then converted to the 0 − 1 range within each species using the following formula:

(target value − minimum value)/ (maximum value − minimum value).

Figures were generated by iTol [36]; the expression values are listed in S4 Table.

### Conserved structure prediction

The Multiple Em for Motif Elicitation (MEME) program [37] was used for the analysis of conserved motifs across all types of PTs or within one type. It was used in the zero-or-one-occurrence-per-sequence (zoops) mode to find motifs with a width ranging from 20 to 50.

Transmembrane helices in proteins were predicted using a website version of TransMembrane prediction using Hidden Markov Models (TMHMM) (http://www.cbs.dtu.dk/services/TMHMM/).

### Pairwise genetic distance estimation

A pairwise genetic distance matrix was calculated in MEGA with a p-distance model. Then, Origin software (OriginLab Corporation) was used to calculate the discrete frequency and create the frequency distribution graphs.

## Results

### Phosphate identification and copy number variation

By searching the genomes of 40 organisms from Bacteriophyta (*Escherichia coli*, *Bacillus megaterium*, *Pseudomonas fluorescens, Streptomyces lavendula*), Eumycota (*Aspergillus flavus*, *Penicillium steckii*, *Saccharomyces cerevisiae*, *Glomus cerebriforme*), Algae (*Chlamydomonas reinhardtii*, *Micromonas pusilla*, *Klebsormidium nitens*, *Chondrus crispus*), Eryophyta (*Physcomitrella patens*, *Marchantia polymorpha*), Pteridophyta (*Selaginella moellendorffii*), basal angiosperm (*Amborella trichopoda*), Monocotyledoneae (*Oryza sativa*, *Leersia perrieri*, *Aegilops tauschii*, *Triticum urartu*, *Spirodela polyrhiza*, *Zostera marina*, *Dendrobium officinale*, *Phalaenopsis equestris*), and Eudicotyledoneae (*Aquilegia coerulea*, *Vitis vinifera*, *Populus trichocarpa*, *Salix purpurea*, *Arabidopsis thaliana*, *Brassica oleracea*, *Brassica rapa*, *Glycine max*, *Cucumis sativus*, *Cucumis melo*, *Citrullus lanatus*, *Ipomoea nil*, *Capsicum annuum*, *Solanum lycopersicum*, *Solanum tuberosum*) and using *Homo sapiens* as an outgroup, 564 PHT1s, 94 PHT2s, 134 PHT3s, 228 PHT4s, 116 G3Pps, 546 pPTs, and 480 SPX-domain PTs were identified (S3 Table).

Copy number variation of each PT type in these genomes was determined (Table 1). In higher plants, the copy number of all kinds of PTs remained in a relatively stable range. PHT1, pPT, and SPX-domain groups had more members than others. The presence or copy number of different PT types varied among taxa. For example, under a BLAST threshold of 1e-5, there were no PHT3s, SPX-domain PTs, or pPTs found in four prokaryote genomes. Moreover, PHT1s showed a lower copy number or were absent in algal taxa, whereas a large copy number of PHT2s was found in *M. polymorpha*. This finding suggests different times of origin of the PT types. Furthermore, every PT type showed hits in *H. sapiens*, implying a very early origin of the common ancestor of animals and plants.

**Table 1:** Copy number variation of seven types of phosphate transporter proteins.

species name	short name	PHT1-hits	PHT1	PHT2-hits	PHT2	PHT3-hits	PHT3	PHT4-hits	PHT4	G3Pp-hits	G3Pp	pPT-hits	pPT	SPX-hits	SPX
**Bacteriophyta**															
*Escherichia coli*	Esco	8	3	2	2	0	0	6	6	4	4	0	0	0	0
*Bacillus megaterium*	Bame	6	3	1	1	0	0	6	6	1	1	0	0	0	0
*Pseudomonas fluorescens*	Psfl	6	2	1	1	0	0	14	14	7	5	0	0	0	0
*Streptomyces lavendula*	Stla	2	1	2	2	0	0	2	2	0	0	0	0	0	0
**Eumycota**															
*Asperigillus flavus*	Asfl	19	15	4	4	4	4	5	5	3	2	3	2	5	2
*Penicillium steckii*	Pest	25	22	2	2	2	2	3	3	2	2	3	2	6	5
*Saccharomyces cerevisiae*	Sace	8	8	1	1	2	2	0	0	0	0	1	1	6	5
*Glomus cerebriforme*	Glce	11	4	2	2	2	2	2	2	2	2	4	4	10	7
**Algae**															
*Chlamydomonas reinhardtii*	Chre	4	0	12	11	1	1	10	9	3	3	11	9	4	2
*Micromonas pusilla*	Mipu	1	1	1	1	1	1	5	5	3	2	5	5	4	1
*Klebsormidium flaccidum*	Klfl	5	1	9	6	2	2	5	5	1	1	11	11	6	5
*Chondrus crispus*	Chcr	0	0	3	3	2	2	4	3	2	2	7	4	1	1
**Bryophyta**															
*Physcomitrella patens*	Phpa	14	8	5	3	9	8	10	3	3	2	23	23	23	15
*Marchantia polymorpha*	Mapo	8	5	24	21	4	4	4	4	1	1	8	8	12	9
**Pteridophyta**															
*Selaginella moelledorffii*	Semo	13	13	2	2	4	4	3	3	3	3	16	13	22	14
**basal angiosperm**															
*Amborella trichopoda*	Amtr	20	13	2	1	3	3	6	6	3	3	12	12	12	10
**Monocotyledoneae**															
*Oryza sativa*	Orsa	24	20	1	1	6	5	7	6	4	4	21	20	15	14
*Leersia perrieri*	Lepe	25	20	1	1	5	5	7	6	5	2	17	16	16	15
*Aegilops tanschii*	Aeta	18	8	1	1	5	5	6	5	5	3	14	9	17	13
*Triticum urartu*	Trur	21	10	1	1	6	5	8	6	3	1	15	11	14	8
*Spirodela polyrhiza*	Sppo	9	7	1	1	4	4	8	6	4	4	14	11	9	6
*Zostera marina*	Zoma	15	12	1	1	2	2	4	4	4	3	21	21	11	8
*Dendrobium officinale*	Deof	15	10	1	1	6	4	10	4	6	1	25	15	17	7
*Phalaenopsis equestris*	Pheq	9	8	1	1	3	3	7	6	4	3	19	18	14	9
**Eudicotyledoneae**															
*Aquilegia coerulea*	Aqco	20	18	2	1	3	3	8	6	4	4	15	13	13	12
*Vitis vinifera*	Vivi	20	18	1	1	5	4	8	6	3	3	20	19	20	16
*Populus trichocarpa*	Potr	37	32	3	2	6	6	9	8	6	4	28	28	35	26
*Salix purpurea*	Sapu	25	24	2	2	7	7	10	10	6	5	22	21	45	31
*Arabidopsis thaliana*	Arth	28	24	1	1	3	3	6	6	7	5	21	19	22	20
*Brassica oleracea*	Brra	46	39	2	2	6	5	8	4	10	6	32	31	43	39
*Brassica rapa*	Brol	64	39	2	2	6	5	7	7	7	6	31	29	52	36
*Glycine max*	Glma	47	46	2	2	9	6	14	12	6	6	48	45	45	34
*Cucumis sativus*	Cusa	17	16	1	1	4	3	6	6	2	2	14	14	18	15
*Cucumis melo*	Cume	15	15	1	1	4	4	6	6	2	2	16	16	16	15
*Citrullus lanatus*	Cila	21	19	1	1	4	4	6	5	2	2	16	13	19	15
*Ipomoea nil*	Ipni	24	23	2	2	3	3	9	8	4	4	24	24	24	20
*Capsicum annuum*	Caan	19	17	1	1	5	4	7	6	3	3	21	20	20	16
*Solanum lycopersicum*	Soly	21	19	1	1	4	4	7	6	5	4	21	20	29	16
*Solanum tuberosum*	Sotu	26	20	1	1	4	4	5	5	5	4	17	17	21	12
**Outgroup – Animal kindom**															
*Homo sapiens*	Hosa	5	1	2	2	1	1	10	8	4	2	4	2	3	1

Footnotes: The first column of each PT type means copy numbers of all BLASTp hits and the second column means copy numbers of sequences with complete domains.

Genome ploidization caused an increase in the number of chromosomes and genes during evolution. Extra copies of some genes provide an opportunity to evolve additional functions via dosage effects, which helps new species to adapt to their external environments. If genes of a given group are favorable for fitness, more copies are likely to be retained. As occurred in Brassicaceae, after speciation from a common ancestor of *Arabidopsis* and *Brassica*, the ancestor of *B. rapa* and *B. oleracea* experienced a triploidization event [38]. Polyploidy-associated rearrangements, homoeologous exchange, and structural variation further shape gene dosage and diversification in Brassica crops, providing a genomic context for interpreting PT copy number variation in this lineage [39]. The increase in the copy number of PT genes in *B. rapa* and *B. oleracea* compared with *A. thaliana* is equal to the increase in the total number of putative genes (147/88, 169/88 vs. 41019/35386, 59220/35386). In Solanaceae, the ancestor of *S. tuberosum* experienced a diploidization event that did not occur in the ancestor of *S. lycopersicum* [40], and the retention rate was not different for the total number of PTs (79/88 vs. 35119/34727) and each type of PTs. In Cucurbitaceae, the copy number in *C. sativus*, *C. melo* and *C. lanatus* has been maintained at a similar level. These findings suggest a high probability that PT genes experienced no selection bias during the domestication of vegetable crops.

**Fig 1:**
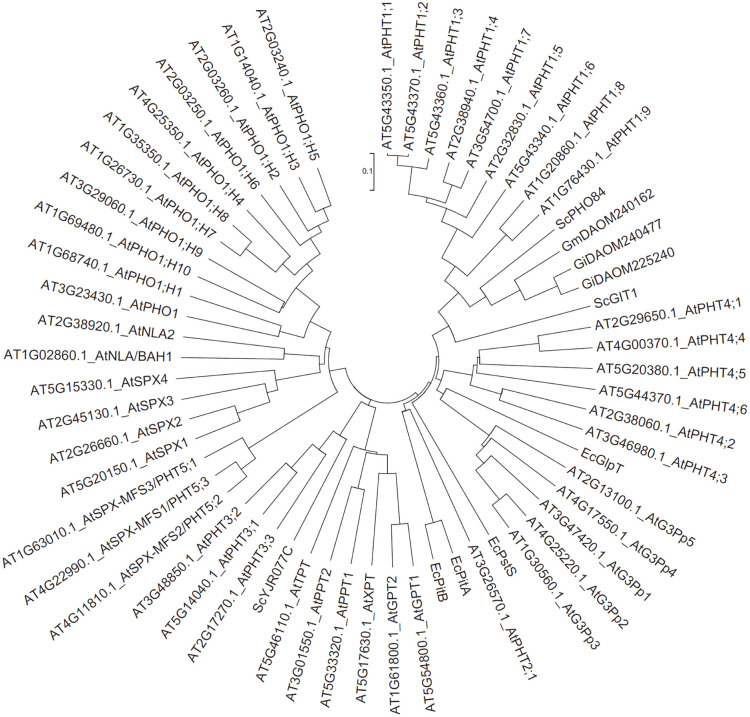
Clustering relationships by the neighbor joining method of seven types of reference phosphate transporter proteins.

To confirm the relationship between different types of PTs, we performed a neighbor-joining (NJ) clustering using the 50 *Arabidopsis* reference sequences together with ten other PTs identified in the earliest studies of *E. coli* (EcGlpT, EcPstS, EcPitA, EcPitB), *S. cerevisiae* (ScPHO84, ScGIT1, ScYJR077C), and arbuscular mycorrhizal fungi (GmDAOM240162, GiDAOM240477, GiDAOM225240) [5,20,28,41–43]. Consistent with previous knowledge, PHT1s PHT2s, PHT3s, PHT4s, G3Pps, pPTs, and SPX-domain PTs respectively clustered together (Fig 1). Most of them have homologs in single-celled organisms (e.g., ScPHO84 for PHT1, EcPitA for PHT2, and ScYJR077C for PHT3), implying an ancestral form of these proteins.

**Fig 2:**
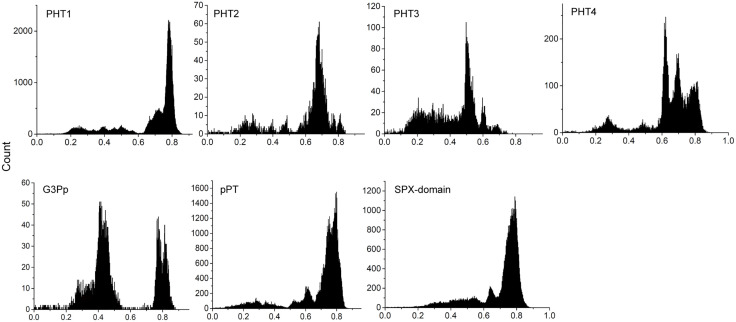
Frequency distribution of pair-wise genetic distances (p-distance) for seven types of phosphate transporter proteins.

Pairwise distances were estimated among all types of PT proteins. Because of the same threshold value when referring to the same database, the frequency distribution of the hit sequences reflects, to some extent, the evolutionary history of the proteins. The results (Fig 2) showed no similar patterns between any two types. This finding suggests an independent origin and diversification of the different kinds of PTs.

### Phylogenetic relationships of phosphate transporters

**Fig 3:**
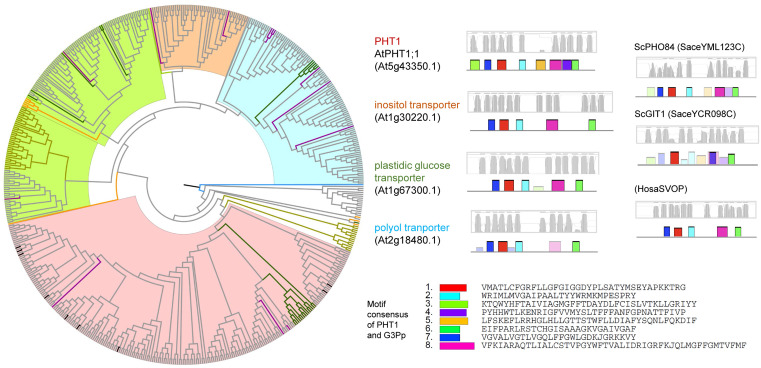
Phylogenetic and structural analysis of PHT1 type of phosphate transporter proteins. Clades are marked with highlights, and the putative transmembrane structures and conserved motifs of representative proteins are shown aside. The sequence names are colored according to their taxa (Bacteriophyta: orange; Eumycota: yellow-green; Algae: light green; Bryophyta and Pteridophyta: dark green; basal angiosperm: purple, Angiosperm: grey), and the reference sequences of Arabidopsis were colored black.

**Fig 4:**
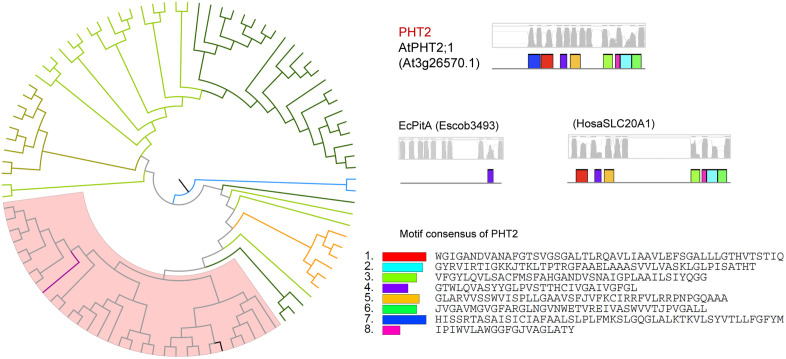
Phylogenetic and structural analysis of PHT2 type of phosphate transporter proteins.

**Fig 5:**
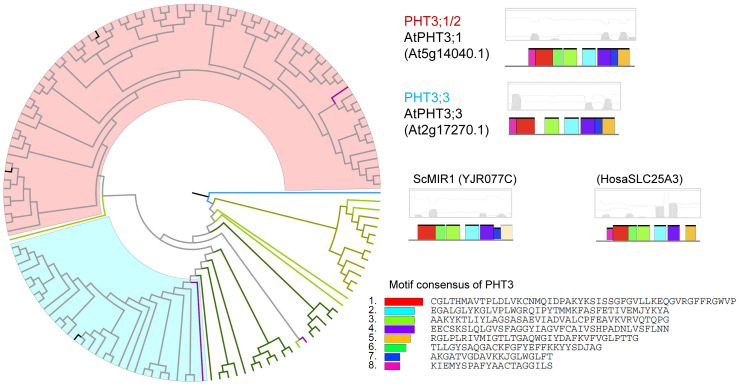
Phylogenetic and structural analysis of PHT3 type of phosphate transporter proteins.

**Fig 6:**
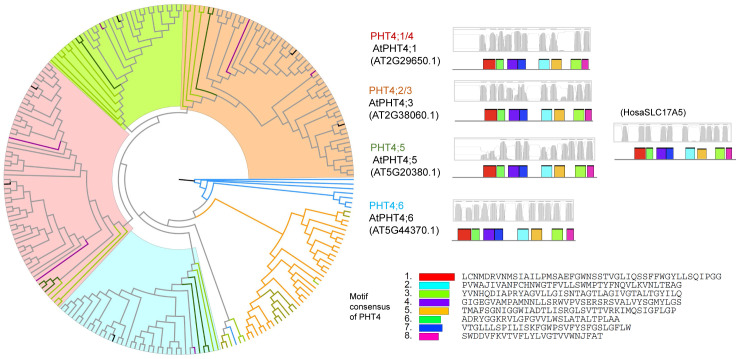
Phylogenetic and structural analysis of PHT4 type of phosphate transporter proteins.

**Fig 7:**
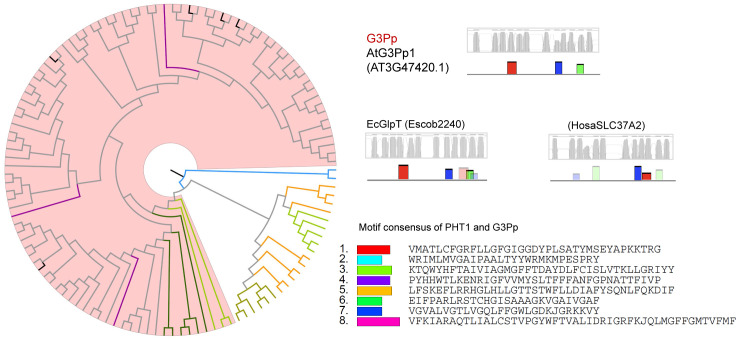
Phylogenetic and structural analysis of G3Pp type of phosphate transporter proteins.

**Fig 8:**
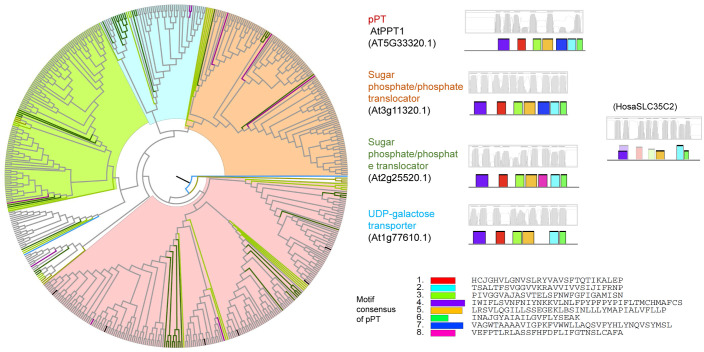
Phylogenetic and structural analysis of pPT type of phosphate transporter proteins.

**Fig 9:**
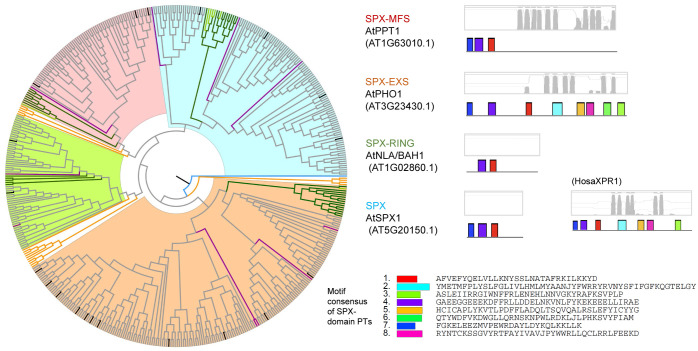
Phylogenetic and structural analysis of SPX-domain type of phosphate transporter proteins.

Owing to their common function of Pi transport, we examined whether PHT1s, PHT2s, PHT3s, PHT4s, G3Pps, pPTs, and SPX-domain PTs showed common motifs. Therefore, all hit sequences of PTs were together analyzed using the MEME program. Except for slight resemblance between PHT1 and G3Pp, no common motifs were found between the various types of PTs. Phylogenetic and structural analysis was performed within each PT type independently (Fig 3–9, S3 Table).

Using *Arabidopsis* PHT1;1–9 as queries, typical PHT1 proteins, along with three homologous clades were searched. Within the typical PHT1 clade (Fig 3, the red clade, S3 Table), PHT1;1–7 were clustered together, whereas PHT1;8–9 were in an adjacent sub-clade. By annotation, other clades were identified to encode potential inositol transporter (yellow clade), polyol transporter (blue clade), and plastidic glucose transporter (green clade). The similarity between these proteins and the typical PHT1 was 22–28%, whereas it was 51–94% within PHT1s. ScPHO84, ScGIT1, and the human homolog SVOP were also included in this type, with 34%, 22%, and 22% similarity with *Arabidopsis* PHT1, respectively. All proteins were predicted to have 11–12 transmembrane domains. In contrast to the large number of PHT1s in angiosperm plants, PHT1s were absent or rarely found in algae, indicating different mechanisms of P transport between these two taxa.

PHT2s from angiosperm genomes were clustered in one clade, distinguished clearly from their homologs in other taxa (Fig 4). EcPitA and EcPitB were included in this type, with 32% and 35% similarity to AtPHT2;1; however, both of them had fewer transmembrane domains than AtPHT2;1. It seems that a copy number expansion occurred in *M. polymorpha*, which likely increased its adaptability in its habitat.

PHT3s were clustered into two clades, PHT3;1/2 and PHT3;3 (Fig 5). ScMIR1 localized to the mitochondrial inner membrane was included in this type, with 34–41% similarity to plant PHT3s; however, no significant transmembrane domain was found in those proteins.

PHT4s were clustered into four clades: PHT4;1/4, PHT4;2/3, PHT4;5, and PHT4;6 (Fig 6). Members of the human SLC17A family were included in this type; they shared 24–39% similarity with plant PHT4s. Every clade included algal homologs, indicating that the diversity of plant PHT4s was established when algae were formed. PHT4;1–5 and SLC17A have 7–10 transmembrane domains, whereas PHT4;6 have 12 transmembrane domains. It is known that PHT4;1–5 is localized to plastid envelope and PHT4;6 to the Golgi apparatus [7]; the difference in the number of transmembrane domains may be the cause of different subcellular locations of proteins among clades.

Plant G3Pp proteins clustered together with their homologs in human (Fig 7), sharing 39–49% similarity, which implies a more conserved function of this type of PTs. EcGlpT belonged to this type, showing 22–25% similarity with plant G3Pps. However, the yeast ScGIT1 (SaceYCR098C), which takes up GroPIns in the medium, was not identified with the G3Pp queries, but with PHT1 queries. In addition, G3Pp proteins possessed motifs similar to the N-terminal motifs of PHT1.

A previous study classified pPTs into four sub-types (TPT, PPT, GPT, and XPT) according to their substrate specificity [44]. In the present study, they were grouped into one clade (red clade) (Fig 8). Additionally, three groups of homologs were identified. They had the potential functions of sugar phosphate/phosphate transporter (yellow clade), UDP-galactose transporter (blue clade), and another sugar phosphate/phosphate transporter (green clade). The similarity shared by them with pPTs was 22–29%. The diversity of the sub-clades originated in algae.

PTs with an SPX domain were previously classified into SPX, SPX-MFS, SPX-EXS, and SPX-RING. In the present study, this classification was confirmed (Fig 9). Human SPX-domain protein showed 24–30% similarity to plant SPX proteins. SPX-MFS and SPX-EXS had six or ten transmembrane domains, whereas SPX and SPX-RING had none.

### Tissue-specific and P starvation-induced expression patterns of various phosphate transporter proteins

**Fig 10:**
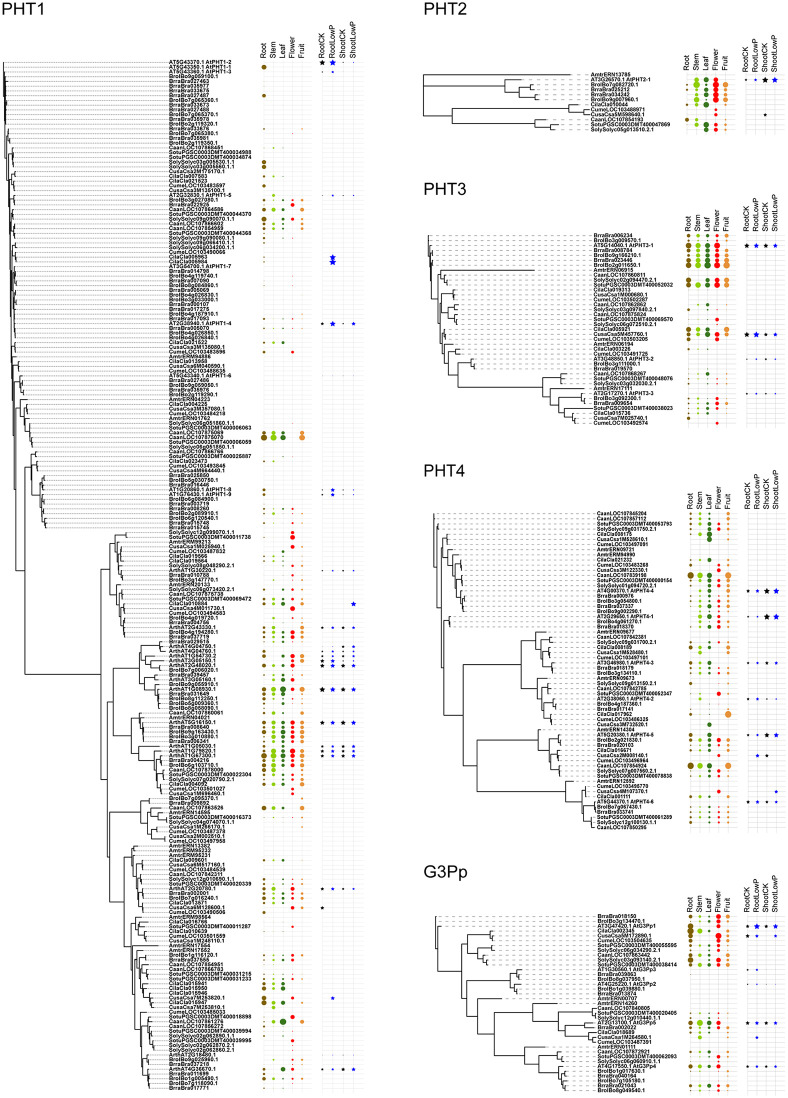
Normalized gene expression levels of PHT1, PHT2, PHT3, PHT4, and G3Pp type of phosphate transporter proteins. The area of the colored circles or stars indicates the relative expression level within a genome. Spaces without colored shapes represent a missing value.

**Fig 11:**
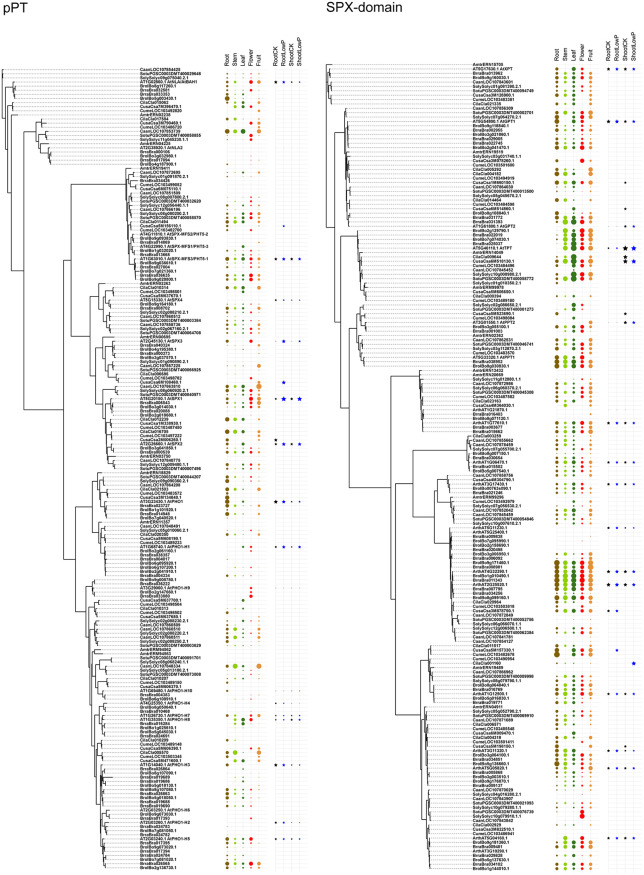
Normalized gene expression levels of pPT and SPX-domain type of phosphate transporter proteins. The area of the colored circles or stars indicates the relative expression level within a genome. Spaces without colored shapes represent a missing value.

Ten genomes were selected for transcriptional expression analysis, including *A. trichopoda*, *A. thaliana*, and eight globally cultivated vegetable species (two brassica, three solanaceous, and three gourd vegetables). RNA-seq transcriptomes were downloaded from public databases (S1 Table). Datasets for five tissues (root, stem, leaf, flower, and fruit) were obtained from *A. thaliana*, *B. oleracea*, *B. rapa*, *C. sativus*, *C. melo*, *C. lanatus*, *C. annuum*, *S. lycopersicum*, and *S. tuberosum*. Datasets for Pi starvation treatments (-Pi, + Pi) were obtained from *A. thaliana*, *C. sativus*, and *C. lanatus*. To increase comparability, normalization and 0 − 1 range transformation were performed for each species and dataset (see Materials and methods). The abundance of PT transcripts in five tissues and Pi starvation treatments is presented on the tree in Fig 10–11. As a whole, sequences from different taxonomic categories displayed similar abundance or tissue-specific expression patterns in accordance with the structural similarity reflected by the position on the phylogenetic tree. The finding suggests conserved regulatory mechanisms among selected species. Moreover, some specific mechanisms existed only in some families or species. Despite the interference due to the differences in developmental stage when sampling, the consistency among species may provide reliable deduction.

Among five tissues, the highest expression level of almost all typical PHT1 occurred in roots; the P starvation data suggest that they are induced by low P supply, with more obvious changes in roots than in shoots. Regarding the PHT1-like clades, proteins in the yellow and green clades showed a constitutive expression pattern across tissues and P conditions. In the blue clade, some proteins in Cucurbitaceae expressed preferentially in roots, which was induced by low P. However, limited sources of data lead to a low reliability of the finding.

PHT2s were expressed mainly in the aerial parts of the plant body; PHT3s were constitutively expressed among all tissues; and PHT4s were expressed more abundantly in leaves. These three types did not show an expression pattern significantly induced by P starvation. G3Pps are more abundant in roots and flowers. Some of them may be expressed in response to P starvation.

Of the pPTs, PPT, GPT, and XPT proteins were generally present in low abundance, and TPT proteins were present in high abundance, mainly in aerial tissues. Proteins in the other three homologous clades were constitutively expressed; several copies in Cucurbitaceae in the yellow clade were upregulated under low P.

The SPX-domain PTs showed more complex expression patterns among different tissues and taxa. In the SPX-MFS clade, the known vacuole-localized transporters, the abundance of genes in every taxon was low, and their expression was slightly induced by P deficiency. In the SPX clade, the genes in Brassicaceae and Solanaceae were expressed preferentially in flowers and fruits, with a pattern of P starvation-induced expression in both roots and shoots, whereas the genes in Cucurbitaceae were preferentially expressed in roots and stems. As for SPX-EXS and SPX-RING, it was difficult to identify a general expression pattern among tissues, except for most homologous genes of AtPHO1, which were expressed maximally in roots.

## Discussion

In the present study, we performed a comprehensive analysis of the phylogenetic relationship and transcriptional expression patterns of all seven types of the currently known P transporters. After having evolved from prokaryotic organisms, the eukaryotic cells began to exhibit a high level of subcellular compartmentalization; the endomembrane system enables cells to accumulate nutrients and metabolites. As higher plants evolved, nutrient allocation to different organs emerged, exhibiting characterized tissue-specific regulation. Development of P-efficient germplasm to exploit such systematic allocation between tissues and subcellular compartments is likely a revolutionary approach.

### Origin and diversification of phosphate transporter proteins

Every clade of every PT type had close homologs in algal genomes, indicating a very old origin and diversification of extant proteins. As P participates in every fundamental life process, conserved regions were found to be present in P transporters in plants and animals. On the basis of the clustering relationships (Fig 1, Fig 3-9) and copy number variation (Table 1), which were mutually consistent, the seven PT types were roughly divided into proteins of prokaryotic origin and eukaryotic origin. PHT3s, pPTs, and SPX-domain PTs are of eukaryotic origin; no gene copies corresponding to these proteins were found in prokaryotic genomes. PHT1s, PHT2s, PHT4s, and G3Pps are of prokaryotic origin; they all had hits in bacterial genomes and had a closer relationship with each other than with the other three types.

Remarkably, the yeast ScGIT1 (SaceYCR098C) was identified in BLASTp with the PHT1 queries but not with the G3Pp queries. It was considered that ScGIT1, which takes up GroPIns in the medium, performs a function similar to that of *E. coli* EcGlpT and *A. thaliana* G3Pp that take up G3P. Furthermore, the three homologous clades besides the typical PHT1 included putative polyol, inositol, or plastidic glucose transporters, and they all possessed a part of conserved PHT1 sequences (Fig 3). In addition, G3Pp proteins possessed several common motifs with PHT1 (Fig 7). This finding suggests the possibility that PHT1 and G3Pp proteins were derived from a common ancestor protein with a phosphate ester transporter function.

### Innate responses of plants to P deficiency and potential modification strategies for breeding P-efficient vegetables

When the Pi nutrient state of plants was converted from sufficient to deficient, several responses of Pi uptake and homeostasis were unfolded in the cells, including cytoplasmic Pi decrease, Pi efflux from the vacuole, replacement of phospholipids by galactolipids and sulfolipids, and reduction in intracellular levels of ATP and ADP [9].

PHT1, PHT2, PHT3, and PHT4 proteins, which had the same properties associated with being Pi-H + co-transporters and different subcellular locations, showed diverse transcriptional responses (Fig 10-11). Typical PHT1 proteins are located in the plasma membrane and are evidently induced by Pi starvation, which is consistent with their function as high-affinity transporters. They are also the target of the symbiosis between arbuscular mycorrhizal fungi and plant roots [45]. Mycorrhizal Pi acquisition is embedded in a plant–AMF–microbiome nutrient exchange continuum, in which phosphorus transfer is coordinated with carbon and nitrogen fluxes [46]. As an ideal candidate for genetic engineering, many efforts have been made using the PHT1 family genes; however, Pi toxicity frequently emerged in PHT1 overexpression lines [44], which was likely because of the redundant expression and overabundance of this type of proteins. In addition to transcriptional regulation, PHT1 activity can be modulated by environmental signaling; immune activation directly inhibits PHT1;4-mediated Pi uptake in Arabidopsis roots [47].

PHT2 proteins are located in the chloroplast envelope and expressed constitutively in shoot tissues. Consistent with this, they did not show Pi-induced expression [7].PHT3 proteins, located in the mitochondrial inner membrane, have adapted to their function of ATP production [48]. PHT4 proteins, the substrate of which is not strictly Pi [7], are expressed relatively constantly in every tissue and were not sensitive to Pi availability. It seems that these three types of PTs had no effective response to the moderate stress and are therefore not suitable targets for modification.

G3Pps maintain Pi homeostasis by manipulating the content of G3P from phospholipid breakdown [19]. They are preferentially expressed in roots, and almost all are induced by Pi starvation. As the putative sister lineage of PHT1, they may participate in some cooperative mechanism of Pi uptake and homeostasis in roots; however, further study is required.

pPTs facilitate the counter-exchange of phosphorylated carbon compounds and Pi between plastids and the cytosol, mediating the replacement of Pi in photosynthetic intermediates during P starvation [6,18]. No obvious changes were found in their transcript levels. Similar findings were observed in the other homologous clades of putative UDP-galactose transporters and sugar phosphate transporters. The occasional upregulation to low Pi stress observed in pPT expression was not considered a determinant of P starvation response.

In the SPX-domain PTs, significant structural diversification has produced complex regulation mechanisms. The SPX-MFS subclade proteins were recently identified to act as a vacuolar phosphate transporter for Pi influx to vacuoles. Because vacuoles are the main reserves of nutrients, the dynamics of vacuolar Pi transport during fluctuation should receive more attention. Autophagy provides another layer of Pi homeostasis regulation by modulating PHT1 transporter abundance and whole-plant Pi balance under different Pi regimes [49]. PHO1, an SPX–EXS family phosphate transporter, is predominantly expressed in root stele cells where it mediates Pi efflux into the xylem for root-to-shoot translocation. At the molecular level, structural analysis of AtPHO1;H1 has provided direct insight into SPX–EXS-mediated Pi transport by resolving Pi- and inositol hexakisphosphate-bound conformations of the transporter [50]. Under Pi-deficient conditions, PHO1 accumulation enhances the rate of phosphate loading into the shoot phloem, contributing to plant adaptation to low Pi availability [17,51]. In addition, the SPX-RING protein NLA post-transcriptionally regulates PHT1s, and the SPX protein SPX1/2 acts as a Pi sensor and shows protein–protein interactions with the P-starvation-induced transcription factor PHR1, preventing the transcriptional factor from binding its target promoters [16,52]. All these proteins play an important switch role.

On the basis of the comprehensive analysis, several candidate strategies for creating P-efficient crops were proposed from the perspectives of breeding, cultivation, and genetic engineering. For instance, molecular markers derived from PTs with proper tissue-specific patterns can be used in marker-assisted selection. They can also be used in rootstock cultivar breeding, after which grafting may be used to improve the PUE of the scion. In light of the P-induced expression patterns of most PTs, intermittent application of P fertilizer may be an alternative cultivation method for raising the PUE. In addition, because genetic modification of a single PT gene often leads to unexpected results, targeting some candidate members of PHT1s, G3Pps, and SPX-domain PTs as a whole may be an effective strategy.

## Conclusions

In this study, a comparative analysis of seven types of phosphate transporters was performed in 40 species encompassing prokaryote, animal, and many plant taxa. Analysis from both the phylogenetic and transcriptomic perspective suggested that the seven types of phosphate transporters have a diverse history of origin and diversification and tissue-specific and Pi starvation-related expression patterns. PHT1s, G3Pps, and SPX-domain PTs exhibited potential for utilization. Some functional proteins of these types that are preferentially expressed in roots or vegetative organs under low-Pi conditions could be chosen as candidate PTs in various species. The present findings provide information for further functional analysis of candidate PTs and suggest artificial modification strategies to obtain P-efficient crops.

## Supporting information

S1 Table Genome and transcriptome sources used in the present study. (XLSX)

S2 Table Reference sequences used as queries for genome-wide identification in BLASTp. (XLSX)

S3 Table Identified phosphate transporter proteins. (XLSX)

S4 Table Standardized (0–1 transformation) log2-transformed expression values. (XLSX)

S5 Table Copy number variation of seven types of phosphate transporter proteins. (XLSX)

S1 Fig Phylogenetic tree of PHT1 and homologous proteins. Clades are marked with highlights. The sequence lines and names are colored according to their taxa (Bacteriophyta: orange; Eumycota: yellow-green; Algae: light green; Bryophyta and Pteridophyta: dark green; basal angiosperm: purple, Angiosperm: grey), and the reference sequences of Arabidopsis were colored black.
(PDF)

S2 Fig Phylogenetic tree of PHT2 and homologous proteins. (PDF)

S3 Fig Phylogenetic tree of PHT3 and homologous proteins. (PDF)

S4 Fig Phylogenetic tree of PHT4 and homologous proteins. (PDF)

S5 Fig Phylogenetic tree of G3Pp and homologous proteins. (PDF)

S6 Fig Phylogenetic tree of pPT and homologous proteins. (PDF)

S7 Fig Phylogenetic tree of SPX-domain PTs and homologous proteins. (PDF)
