# Supplementary figures and images for: Comparative analysis of seven types of phosphate transporters in forty species from the phylogenetic and transcriptomic perspective
Source: PLoS One. 2026 May 21;21(5):e0349574. doi: 10.1371/journal.pone.0349574 (PMC13193548; doi:10.1371/journal.pone.0349574)

Supplementary Figure 1

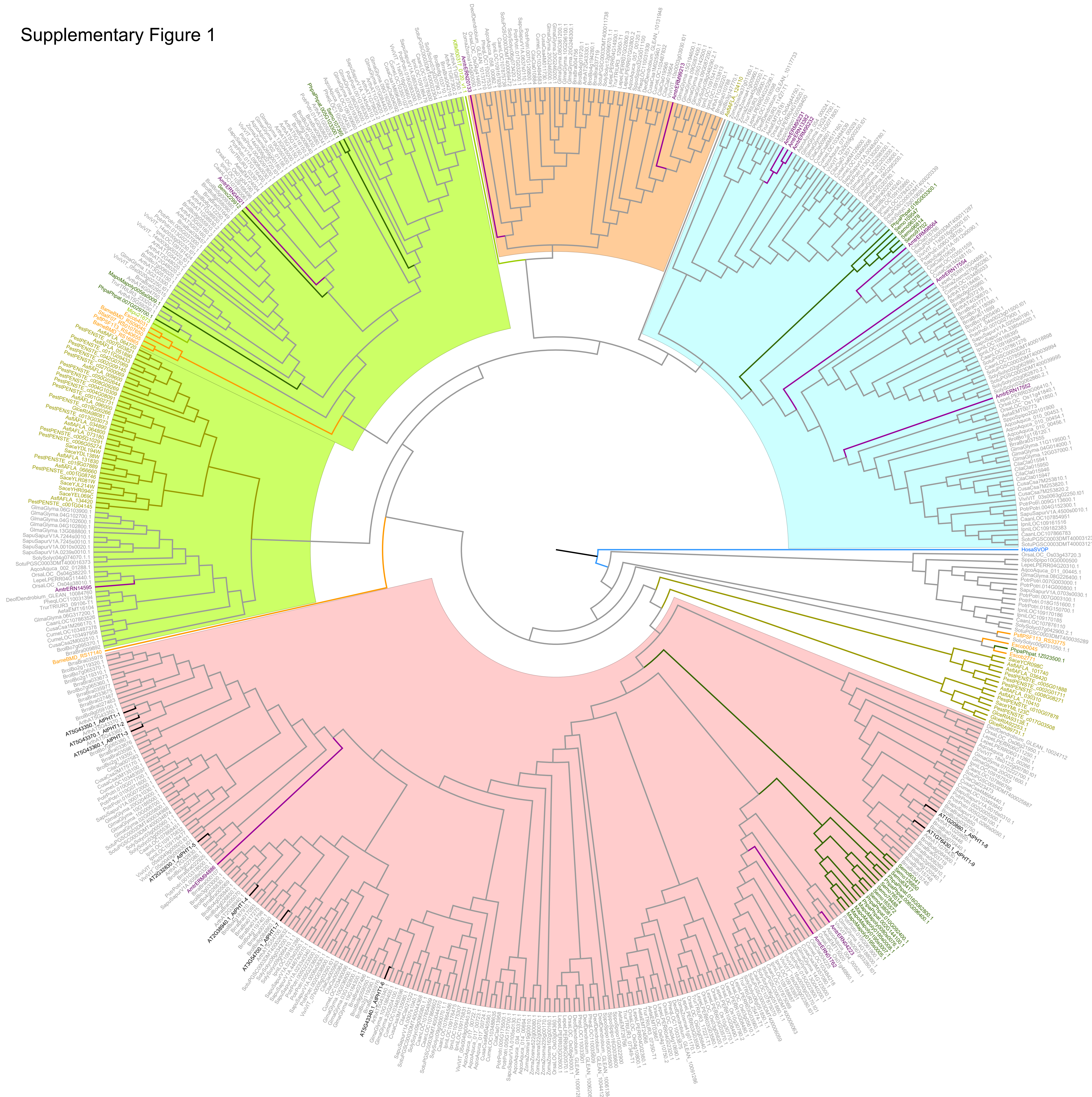

Supplement: S1 Fig — Clades are marked with highlights. The sequence lines and names are colored according to their taxa (Bacteriophyta: orange; Eumycota: yellow-green; Algae: light green; Bryophyta and Pteridophyta: dark green; basal angiosperm: purple, Angiosperm: grey), and the reference sequences of Arabidopsis were colored black. (PDF) [file pone.0349574.s006.pdf]

Supplementary Figure 2

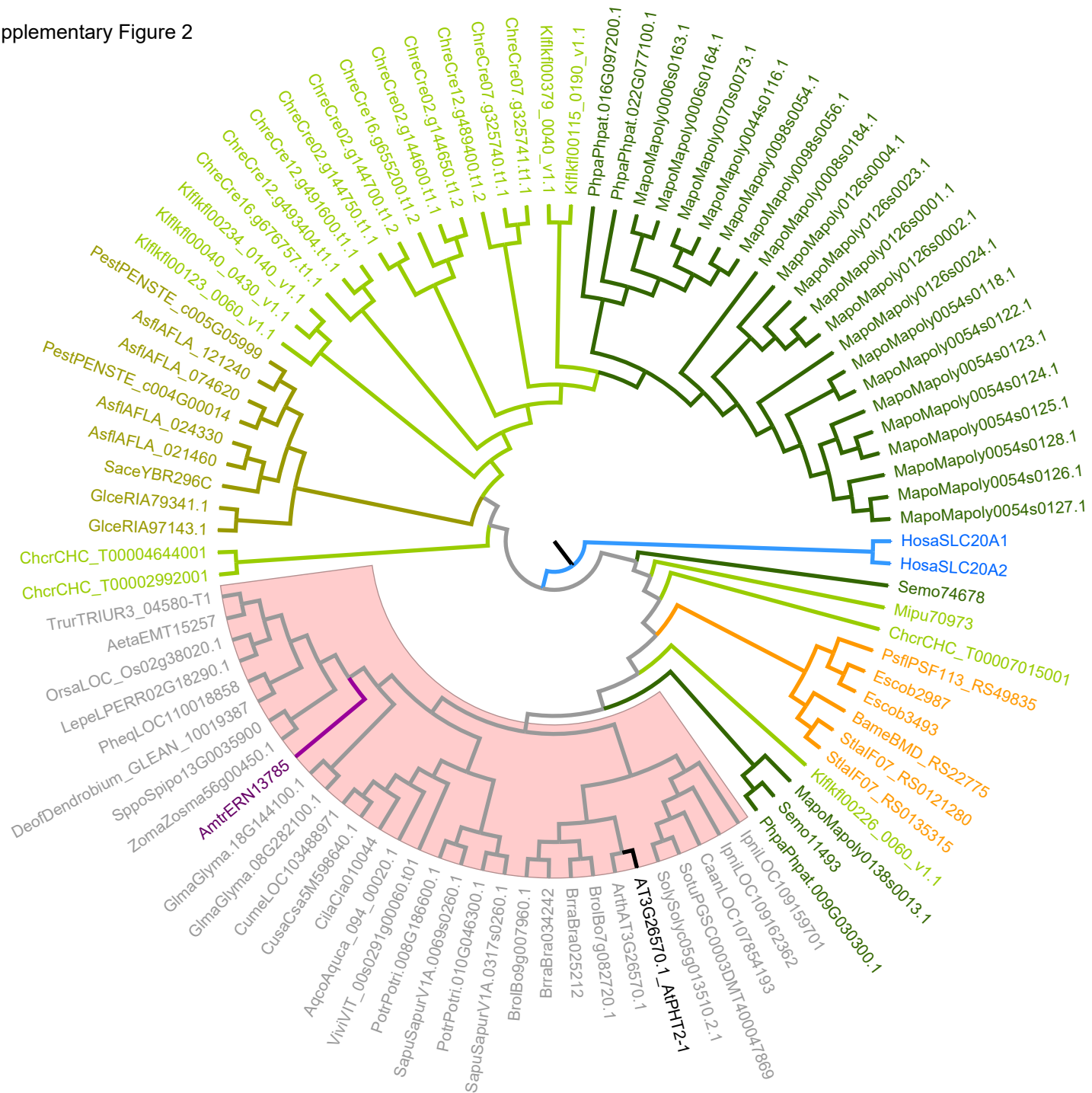

Supplement: S2 Fig — (PDF) [file pone.0349574.s007.pdf]

Supplementary Figure 3

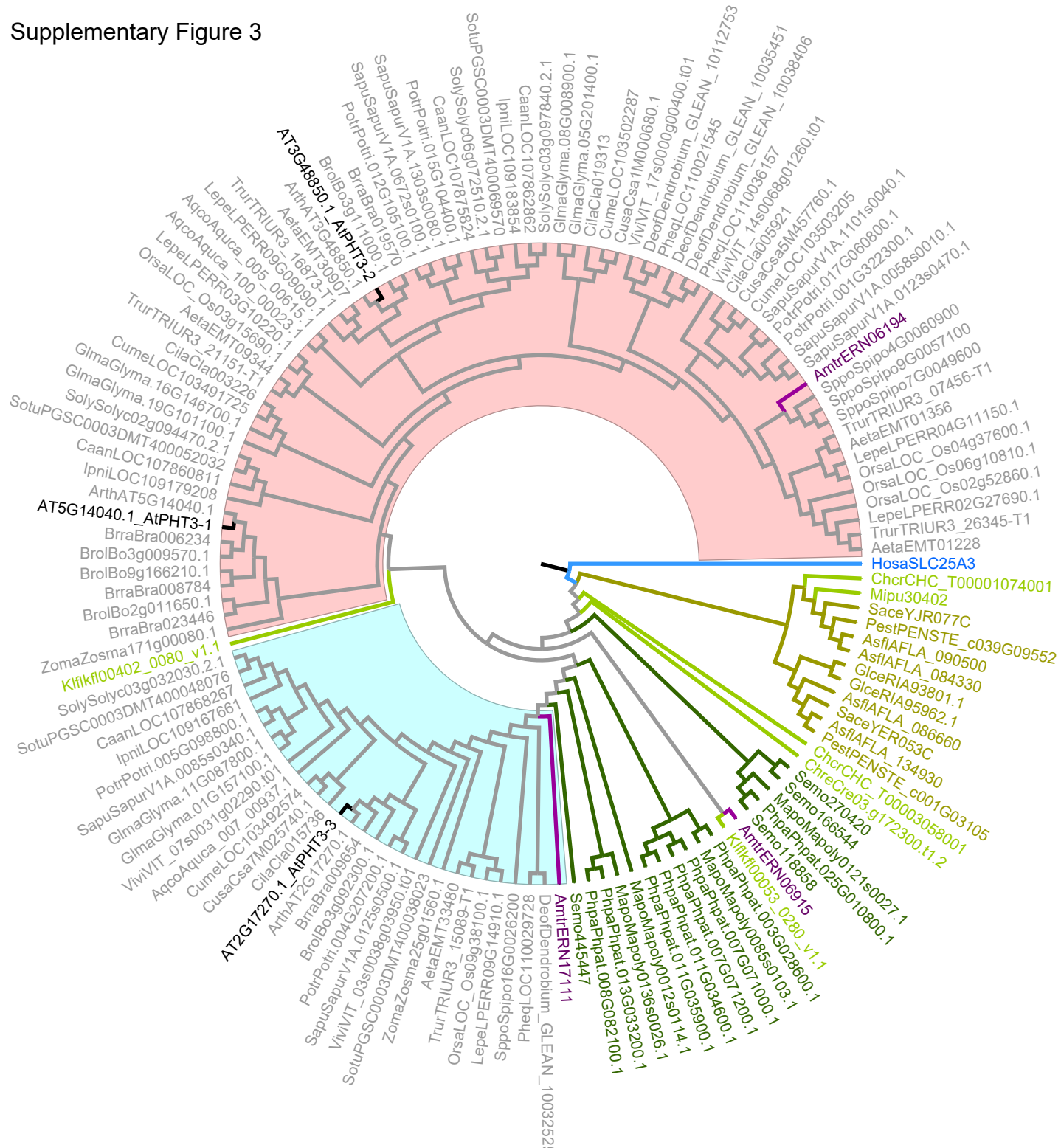

Supplement: S3 Fig — (PDF) [file pone.0349574.s008.pdf]

Supplementary Figure 4

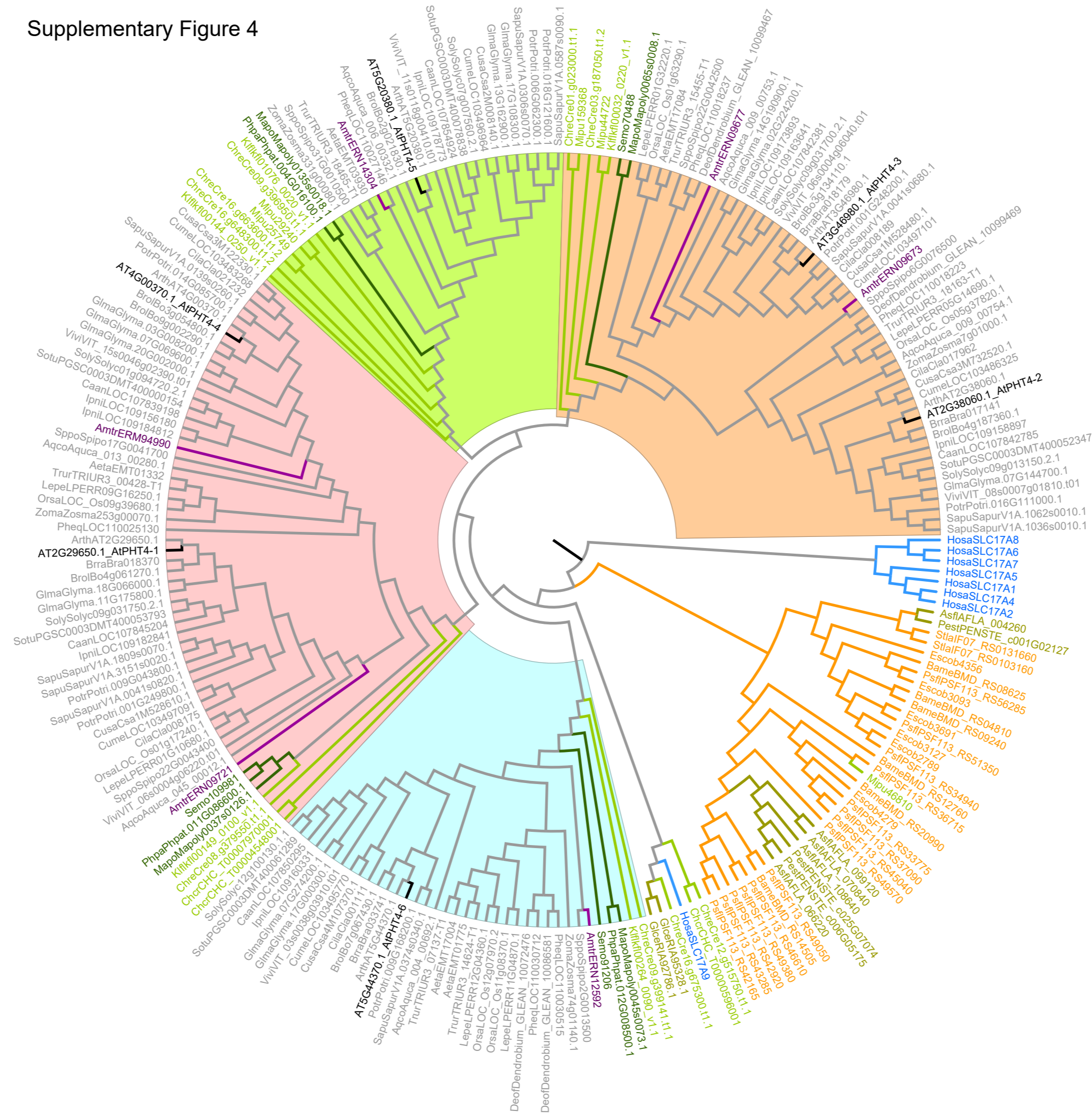

Supplement: S4 Fig — (PDF) [file pone.0349574.s009.pdf]

Supplementary Figure 5

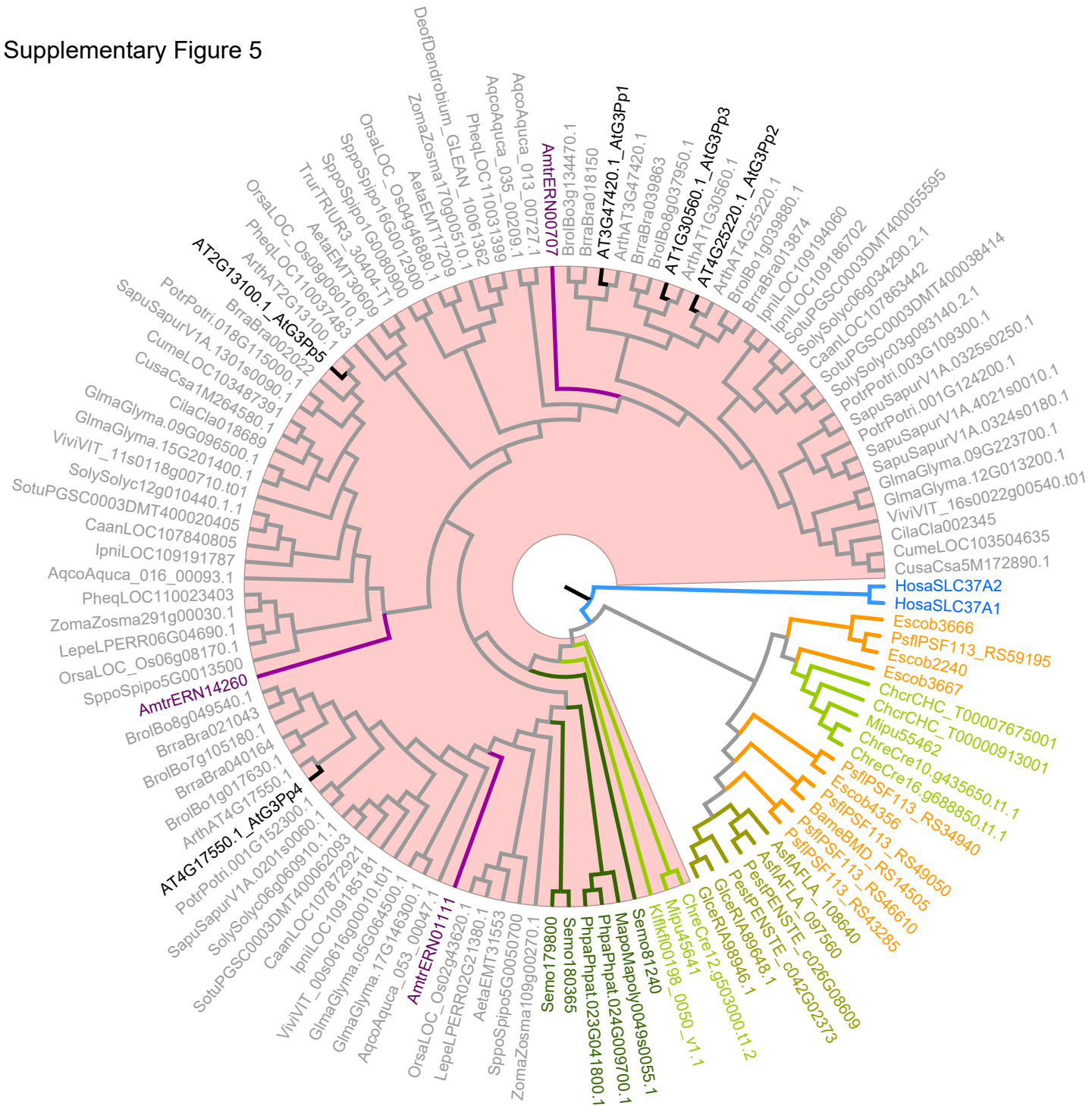

Supplement: S5 Fig — (PDF) [file pone.0349574.s010.pdf]

Supplementary Figure 6

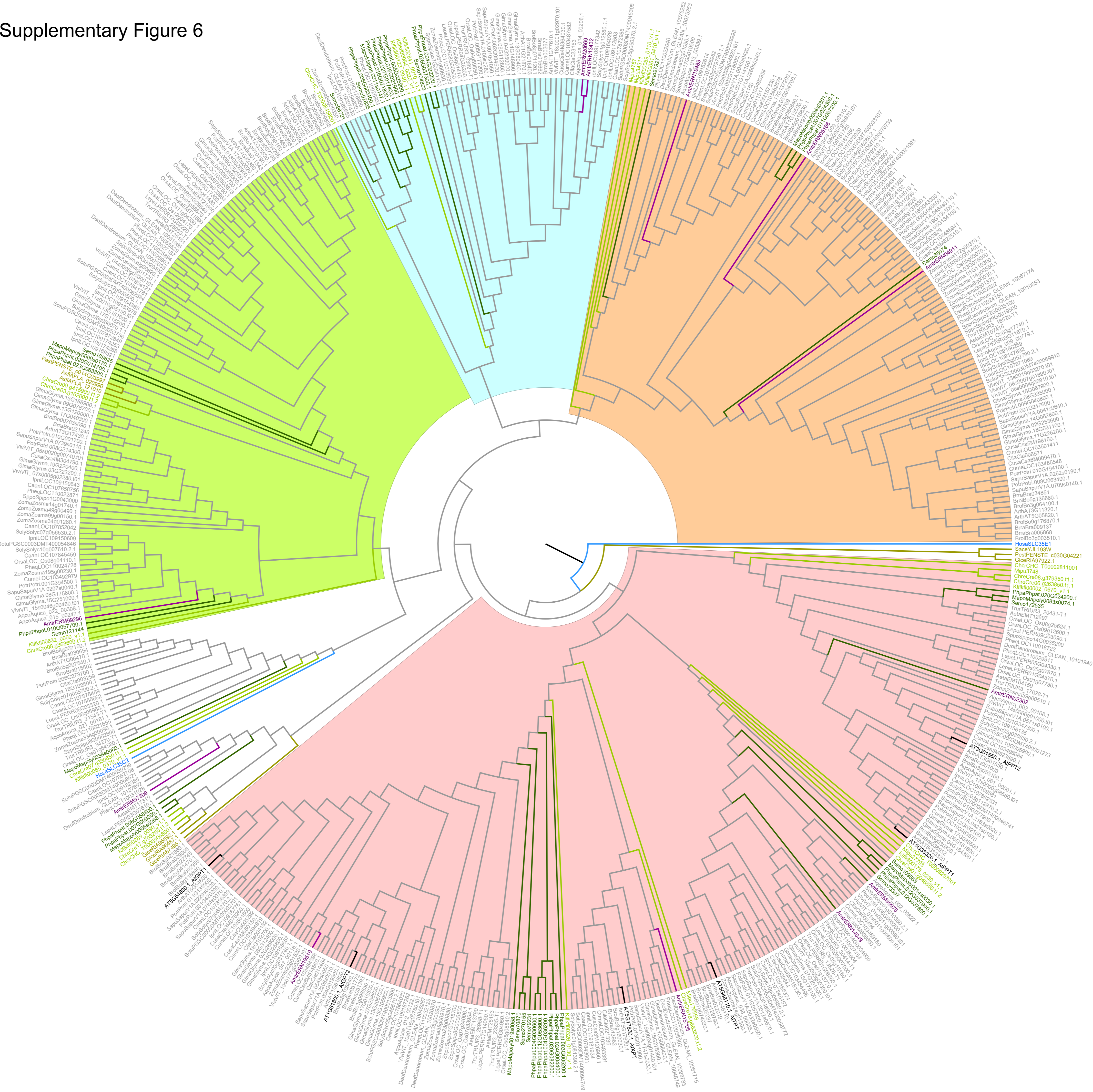

Supplement: S6 Fig — (PDF) [file pone.0349574.s011.pdf]

Supplementary Figure 7

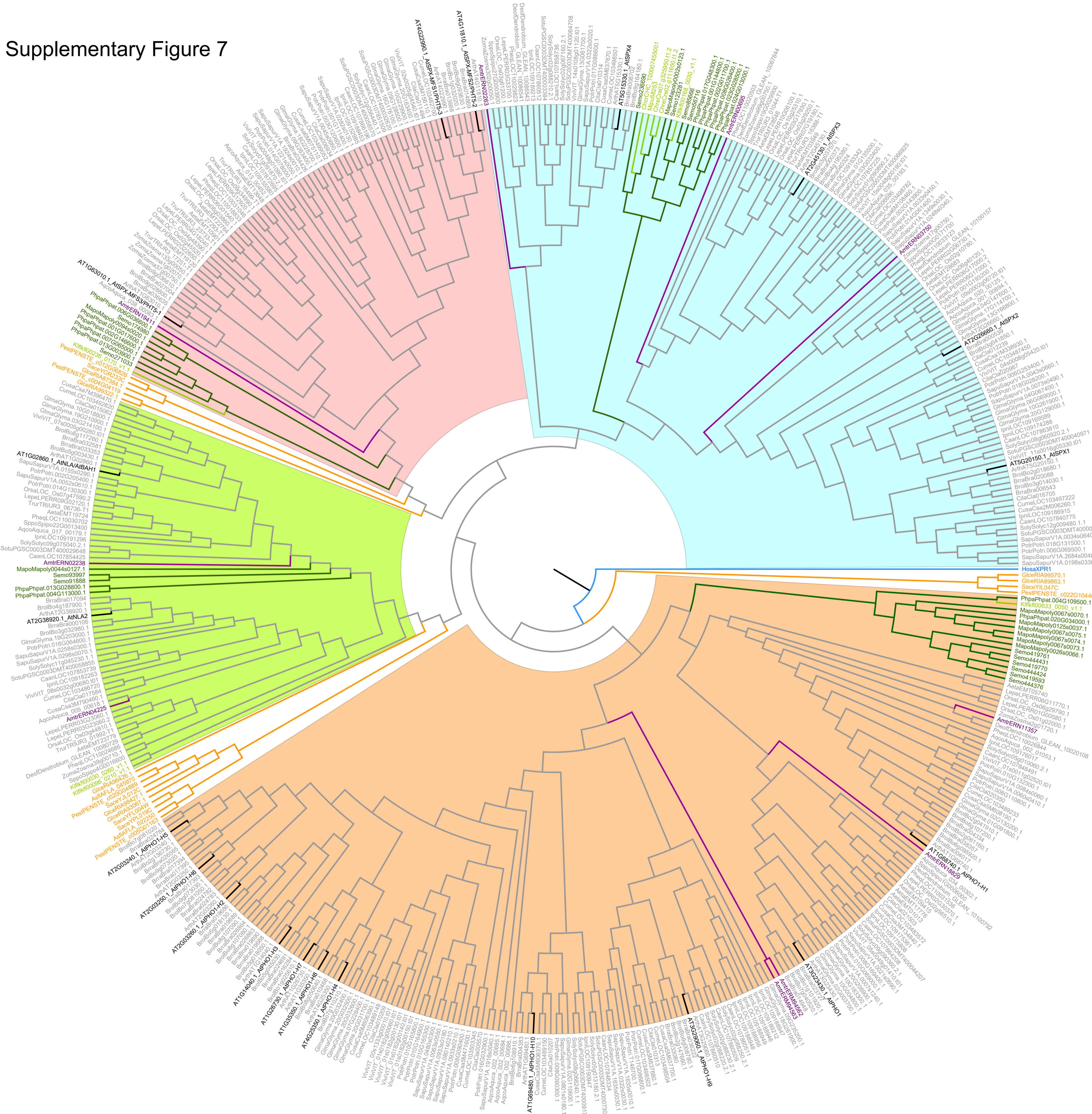

Supplement: S7 Fig — (PDF) [file pone.0349574.s012.pdf]
